# Lake Erie Medical Society—A Case of Hydrocele Treated with Ammonio Ferric Alum

**Published:** 1867-06

**Authors:** H. M. Rogers

**Affiliations:** Dunkirk


ART. II.—*Lake Erie Medical Society—A case of Hydrocele treated with Ammonio Ferric Alum.* BY H. M. ROGERS, M. D., *Dunkirk.*

It will be remembered by those members of the Society who were present at the meeting in Dunkirk in May last, that I reported a case of hydrocele, successfully treated with a remedy comparatively new to the profession, and which had never before been applied in that disease. I refer to the ammonio ferric alum.—Permit me to call your attention to another and a successful case treated with the same remedy.

In the present instance the tumor was one of unusual dimensions, and I think will be regarded as unique in some of its features. The following is its history:

The patient, J. R., is a German, 45 years of age, healthy, and of good constitution. About nine years since, while a soldier in the revolution of 1848, he received a kick from a comrade upon the scrotum, which resulted in inflammation, followed by hydrocele. This hydrocele continued for many years, small in size, and with but little inconvenience or pain, and but little was done in form of treatment. In the year 1864, while working in a brick-yard, he had the misfortune to be much injured in the back and about the perineum and scrotum, by the fall of a pile of bricks. This accident was followed by an increase in the size of the tumor, and the patient came under the professional care of the late Dr. C. K. Irwin, of Dunkirk. Dr. I. in 1865 drew off the contents, amounting to three quarts of bloody serum; since which time nothing has been done in the way of treatment until he came under my charge in February of the present year. On presenting himself at my office I found the dimensions of the scrotum to be, from perineum to the pubic arch 15 inches, and the circumference 17 inches, and upon drawing off the contents the amount removed was forty-eight ounces.

It will be remembered that in my previous paper I remarked, that where the tumor was very large it was my custom to make a preliminary operation by drawing off the contents and permitting the case to remain without other treatment than the suspensory bandage, until the secretion again reached a point where it would be safe to use the trocar. The object, as mentioned, is to permit the distended, morbid, and patulous, tunica vaginilis testis, to resume in a measure its healthy tone, which is conducive to the operation. In the present case the sac filled rapidly, and at the end of three weeks and a half I was again warranted in introducing the trocar and canula, and the quantity of serum removed was something over three ounces. I then introduced a solution of ammonio ferric alum, twelve grains to the ounce of water, and permitted it to remain several minutes. Unlike the case previously reported, this injection was followed by some pain and the usual general symptoms, but very much less in intensity than usually follow the exhibition of the ordinary remedies. The patient was in a condition after a few days to resume his accustomed labors. At the present time, now nearly eight weeks since the operation, there is no appearance of effusion, and with the exception of some thickening of the scrotum consequent upon contraction of its walls, the scrotum and testes appear perfectly normal.

A very singular and perhaps unheard of feature in the case is, that all external appearances of a penis have, within the past three years entirely disappeared, having retracted and the integuments usually covering the parts have been appropriated by the walls of the scrotal sac. His present appearance is as one having been born without that organ. The aperture through which urination takes place can only be discovered by the closest inspection, and the presence of a glans penis and the vestage of a corpus cavernosum can only be found by careful manipulation.

For the benefit of those present who are not acquainted with the particulars of the other case referred to I would say, that it was one of many years' standing, and which had been treated in the usual manner, by injections of tinc. iodine of various degrees of strength, and of Port wine, and by the removal of a portion of the scrotum and tunica vaginalis testis, but without success. It yielded with one application of the ammonio ferric alum, and the cure was painless, immediate and permanent. These two cases have been well marked, and I may say unusual ones, and the success positive; yet, it will require very many other trials to establish its real value. It is to be hoped that if opportunity offers, this remedy may be repeatedly tested and the results reported.

